# Impact of heavy rains of 2018 in western Japan: disaster-induced health outcomes among the population of Innoshima Island

**DOI:** 10.1016/j.heliyon.2020.e03942

**Published:** 2020-05-25

**Authors:** Srinivas Bandaru, Shunji Sano, Yurika Shimizu, Yuka Seki, Yoshikazu Okano, Tamaki Sasaki, Hideho Wada, Takemi Otsuki, Tatsuo Ito

**Affiliations:** a Department of Public Health, Okayama University Graduate School of Medicine, Dentistry, and Pharmaceutical Sciences, Okayama, Japan; b Department of Surgery, Division of Pediatric Cardiothoracic Surgery, University of California San Francisco, San Francisco, CA, USA; c Department of Pathophysiology - Periodontal Science, Okayama University Graduate School of Medicine, Dentistry, and Pharmaceutical Sciences, Okayama, Japan; d Hitz Hitachi Zosen Health Insurance Association Clinic at Innoshima, Onomichi, Hiroshima, Japan; e Innoshima General Hospital, Onomichi, Hiroshima, Japan; f Department of Nephrology & Hypertension, Kawasaki Medical School, Kurashiki, Okayama, Japan; g Department of Hematology, Kawasaki Medical School, Kurashiki, Okayama, Japan; h Department of Hygiene, Kawasaki Medical School, Kurashiki, Okayama, Japan

**Keywords:** Epidemiology, Occupational health, Public health, Quality of life, Japan heavy Rain, Water outage, Health impact, Urinary protein, Health checkup

## Abstract

Southwestern Japan suffered its worst rains in 2018 causing floods and mudslides, claiming 225 lives and forcing millions for evacuations. Referred as “Heisei san-jū-nenshichi-gatsugōu”, the disaster was the result of incessant precipitation caused by the interaction of typhoon “Prapiroon” with the seasonal rain front "Baiu". The present epidemiological study aims to investigate disaster-induced health issues in 728 residents of Innoshima island in the Hiroshima Prefecture by comparing their clinical data in pre-disaster (2017) and disaster-hit (2018) years which was obtained from annual health screening. Comparison of data showed a significant increase in the urine protein concentration in victims following the disaster. Probing further into the household conditions, showed that a total of 59,844 households were affected with water outage during the heavy rains, which was accompanied by severe damage of sewerage pipelines with complete recovery process taking two weeks. This two weeks of the crisis forced victims to refrain from using restrooms which in turn led to infrequent urination, thereby explaining the increased urine protein concentration in victims following the disaster. The present study addresses the acute health implications caused by the water crisis and serves as a precautionary measure for disaster management council to provide enhanced aftercare services in victims in further events of natural disasters.

## 1 Introduction

Southwestern Japan suffered its worst disaster in 2018 caused by torrential rainfall that the Country has ever seen in more than three decades [1]. Officially referred as “Heisei san-jū-nenshichi-gatsugōu” (平成30年7月豪雨, “Heavy rain of July, Heisei 30”) by the Japan Meteorological Agency (JMA), the rains resulted in widespread devastating floods and set off landslides killing 225 people while forcing millions to evacuate across 15 prefectures [2, 3, 4, 5]. In the meteorological history of Japan, it remains the deadliest freshwater disaster since the 1982 Nagasaki floods wherein 299 casualties were reported [6].

**Figure 1:**
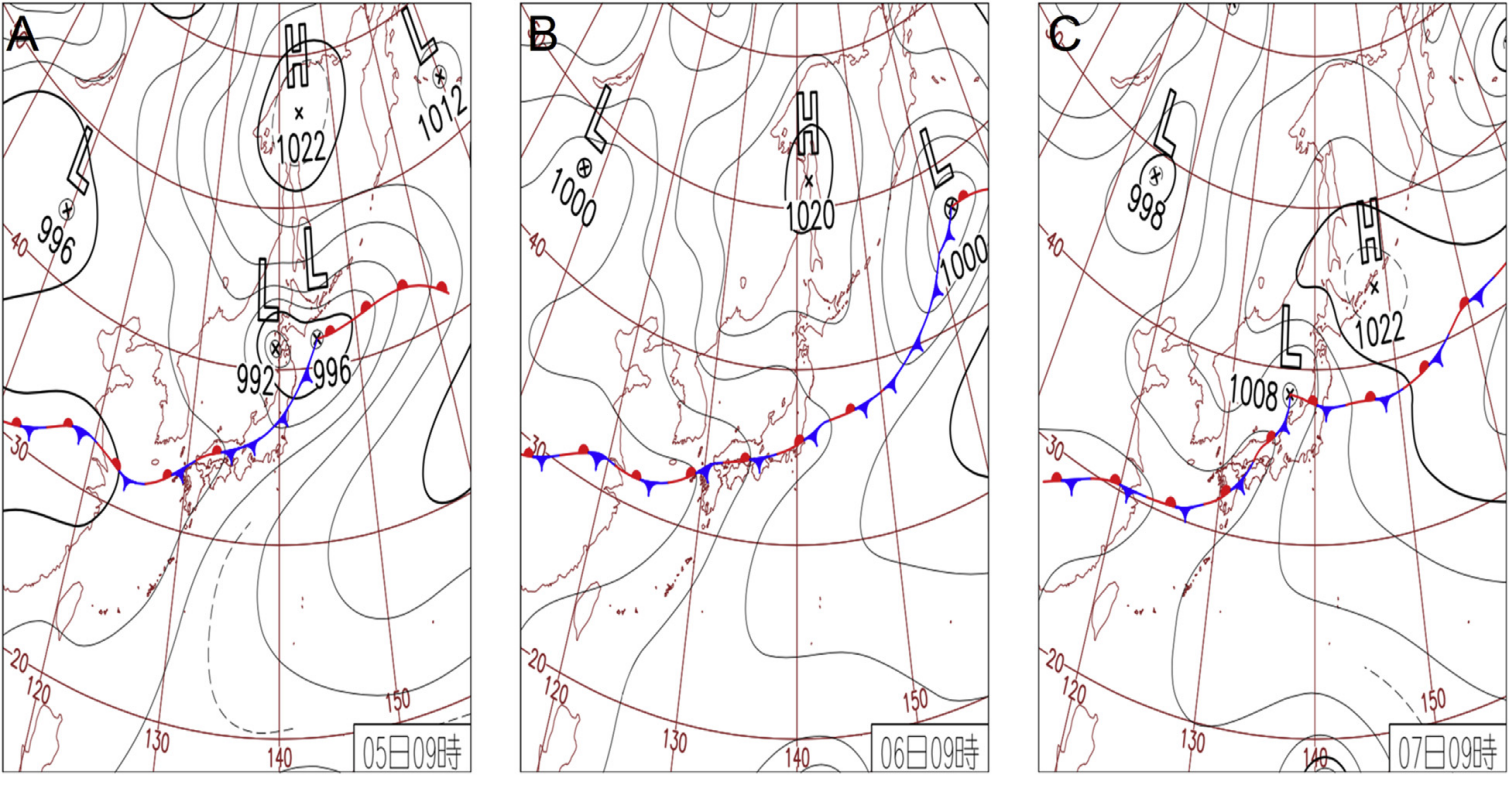
Weather map of three days of heavy rainfall. (A) July 5 2018: Low pressure recorded near Hokkaido. Front extends from northern Japan to southern and western regions carrying warm damp air. Okinawa prefecture receives heavy downpour with 111mm/1h. (B) July 62018: Special warning for heavy rain announced in 8 prefectures of Nagasaki, Saga, Fukuoka, Hiroshima, Okayama, Tottori and Kyoto. Front line activity becomes stagnant around Honshu. It is the first time in the history that Kochi Prefecture receives precipitation of 510.5 mm and that the precipitation of 422.5 mm was recorded in northern Saga prefecture. (C) July 72018: Recorded heavy rains in West Japan and issued special warning in Gifu prefecture. Gifu prefecture's Miboro daily rainfall reaches 330.5 mm, for the first time in history. (Weather information obtained on May 2019 from Japan Meteorological Agency's official homepage; https://www.jma.go.jp/jma/indexe.html).

According to JMA, every year “Baiu”, a seasonal persistent baroclinic rain-front extending from west becomes stationary at a non-tropical low near Hokkaido that usually brings about heavy downpour in the regions under these fronts [2]. The rainfall of 2018 was however unprecedented because the remnants of typhoon “Prapiroon” came along the Baiu front which was further fuelled by warm air from the Pacific Ocean [2]. The synergistic interaction of typhoon and seasonal fronts, steered enhanced precipitation with 10-day incessant rainfall in large swathes of populated areas, accumulating an excess of 400 mm of precipitation (Figure 1). Hiroshima prefecture alone had 1, 243 mudslides, which is far more than the entire nation's total in an average year [7, 8, 9].

The large death toll can principally be attributed to two main reasons. First, people lacked proper awareness of heavy rains and despite advisories to evacuate, (which was, of course, issued too late), some residents ignored the warnings as they did not know how to get to a safe place [10, 11]. Soon after evacuation advisories (from Hiroshima Prefectural Government's headquarters for disaster control) were issued, only 6,000 took shelter out of 1.52 million people [12]. Secondly, Japan being one of the most seismically active countries in the world, people herein follow government-endorsed rescue guidelines for earthquakes associated disasters, nevertheless, hardly have the awareness to rescuing from heavy rain disasters.

The impact of the disaster was so large that the industries, freight services, and railways, all suspended their operations in South-western and central prefectures of the country [13, 14]. The nation sustained tremendous damage to all the major sources of economy, including agriculture, forestry, and fishery amounting to a loss of ¥1.09 trillion (US$9.86 billion) [15]. This sudden shut down in turn grossly came up with a shortage of water, food, and shelter, afflicting people with psychological stress, physical health ailments, and most importantly water-related health issues [16].

The present study aims to investigate disaster-induced health implications in 728 victims of Innoshima (a small island incorporated into Onomichi City in the Hiroshima Prefecture) by a prospective comparison of their clinical data obtained from annual health screening in the pre (2017) and disaster-hit (2018) years.

## 2 Materials and methods

### 2.1 Study population

Ministry of Public Health, Government of Japan, makes a mandatory requirement for its citizens to undergo annual health screening. By making it an annual event, the Government ensures everyone participates in, and medical practitioners address the health concerns of an individual every year. This enables an individual to track their health conditions and monitor physiological changes, both small and major, against the previous year.

The annual health screening was of utmost importance in the present study, as it helped to understand the physiological changes incurred in victims due to heavy rains in 2018 and the magnitude at which these rains worsened their health condition in contrast to the pre-disaster year (2017). In the present investigation, a total of 728 victims were studied for their health effects those mandatorily having registered health check-up data of 2017. It was ascertained that the population recruited herein were victims and physically experienced heavy rains in Innoshima.

Furthermore, it was assured that all the 728 individuals investigated for the health status in the present study were healthy and had no history of any debilitating diseases like cardiovascular diseases, any systemic complications or life-threatening ailments like cancer. Indeed, all the 728 individuals studied herein happen to be regular employees at Innsohima Zosen shipyard which serves as a major organization of employment for the population of Innoshima Island. The rationale behind selecting the subjects from Innoshima Zosen shipyard is for the fact that the shipyard forms the major employment institution for the Innoshima population. In fact, most of the households in Innoshima have a person working in this shipyard, therefore a person screened for health after the disaster may most likely reflect the plight of the health of the other members of the house as well. Therefore, we recruited employees from Innoshima shipyard itself as subjects for our study, which in turn will be representative of the Innoshima's socio-demographics as a whole. Nevertheless, a total of 1884 individuals-majorly the employees of Innsohima Zosen shipyard also underwent annual health check in the pre and disaster-hit years, we, however, narrowed down only to 728 individuals, considering the rest of 1156 out of 1884 individuals had complaints of minor ailments or had a long history of hypertension, respiratory problems, etc, orhave underwent recent hospitalisations within a year of health registration. We intentionally included healthy individuals, so that only disaster-induced physiological outcomes could be assessed, thus ruling out the plausibility of pathology influenced differences in clinical readings in a given individual.

### 2.2 Data collection

Complete information on the health status of victims, including demographic data, was accessed from the clinical reports provided on request by Chugoku Occupational Health Association and SRL, Inc, Japan - the two major public health foundations which conducts annual health screening in Japan. The report registered laboratory investigation values involving routine blood and urine examination, liver function test and kidney function test for both pre (2017) and disaster-hit (2018) years. The annual health screening in 2017 was conducted tentatively between July 11^th^ to 25^th^ and in 2018 from July 19th to September 2nd. Disaster induced physiological changes in victims were assessed by comparing the clinical reports of victims recorded in disaster-hit (2018) to pre-disaster (2017) years.

### 2.3 Ethical considerations

This study protocol was approved by the ethics committee of Okayama University (approval number: 1807-003). Upon the institutional approval, the health screening agencies viz., Chugoku Occupational Health Association and SRL Inc, Japan, along with Hitachi Zosen Health Insurance Association at Innoshima were informed about the prospects of the study. Only upon approval from these associations, the health screening professionals (from Chugoku Occupational Health Association and SRL, Inc,) were directed to orally inform participants and thoroughly explain the study prospects and that their health data will be used for the research investigation. Further, it was also informed to the participants that their names will be kept confidential, and in no respect will be revealed in any circumstances, cases or events and part thereof has not been used for any other study prior to this date. Therefore, only upon the consent of the participant, their data was collected. As their names were withheld, all the 1884 participants, therefore, agreed to join the research (of 1884 participants we selected only 728 individuals considering the rest of them had complaints or history of diseases). The final health data that was provided to us by Chugoku Occupational Health Association and SRL, Inc, had general demographic data along with the clinical data, except for the names of the participants.

### 2.4 Statistical analysis

All the clinical and demographic variables registered for pre-disaster (2017) and disaster-hit (2018) years for an individual was compared by paired student t-test. Two-way Anova was applied in comparison of groups that have been categorized for independent factors. Binary logistic regression was performed to study the multivariate association of the factors which contributed to the disaster-induced deterioration of health by considering year of health screening as a depended variable and the clinical and demographic variables of the individuals corresponding to the year as independent variables. Frequency distribution across the variate was tested for statistical significance with χ2 test (Yates corrected) along with odds ratio at a confidence interval of 95%. Results were considered significant with a cut-off of p-value less than 0.05 (two-tailed).

## 3 Results

### 3.1 Comparative analysis of physiological changes for pre-disaster (2017) and disaster-hit (2018) years

Laboratory investigation values for an individual registered in the pre-disaster (2017) and disaster-hit (2018) yearshealth screening program were compared to assess the disaster-induced physiological changes in victims. As evident from Table 1, a significant difference in the levels of WBC, RBC, Hct, Alb, Uric Acid, HbA1C, and more importantly in the urine protein was observed. These results reasonably indicate that heavy rains brought about significant health deterioration in individuals majorly affecting blood and urine physiology.

**Table 1:** Comparison of epidemiological and physiological variates of victims in pre-disaster (2017) and disaster-hit (2018) years.

	Pre-disaster year (2017)	Disaster-hit year (2018)	p value
	Mean ± SD	Mean ± SD	
Age (years)	49.45 ± 10.19	50.45 ± 10.19	0.06
Height (Cms)	167.92 ± 7.38	168.09 ± 7.45	0.64
Weight (Kg)	68.71 ± 13.28	68.33 ± 13.04	0.61
BMI (cells/μL)	24.30 ± 4.07	24.11 ± 3.99	0.38
WBC (cells/μL)	6.22 ± 1.7	5.99 ± 1.59	<0.05
RBC (cells/μL)	4.62 ± 0.42	4.72 ± 0.44	<0.01
Hb (Gram %)	15.09 ± 1.22	15.05 ± 1.25	0.51
Hct (Gram %)	46.19 ± 3.70	45.43 ± 3.73	<0.01
MCV (fl/cell)	100.01 ± 5.64	96.33 ± 5.43	<0.01
MCH (pg/cell)	32.67 ± 1.94	31.92 ± 1.9	<0.01
MCHC (g/dL)	32.67 ± 0.81	33.14 ± 0.84	<0.01
PLT (Volume %)	23.39 ± 5.84	23.53 ± 5.42	0.62
LDL (mg/dL)	117.60 ± 28.15	119.40 ± 29.21	0.23
HDL (mg/dL)	59.48 ± 15.09	59.99 ± 15.37	0.52
TG (mg/dL)	120.67 ± 94.92	128.82 ± 117.10	0.14
AST (Units/L)	24.11 ± 10.07	25.03 ± 18.35	0.24
ALT (Units/L)	26.13 ± 18.39	28.01 ± 23.40	0.09
ALP (Units/L)	205.78 ± 62.77	208.19 ± 65.25	0.47
γGTP (Units/L)	46.28 ± 60.17	50.68 ± 78.82	0.23
LDH (Units/L)	199.04 ± 35.03	195.7 ± 36.9	0.08
Total Protein (Units/L)	7.37 ± 0.35	7.34 ± 0.37	0.25
Alb (g/dL)	4.61 ± 0.27	4.55 ± 0.27	<0.05
TBil (mg/dl)	0.8 ± 0.33	0.82 ± 0.37	0.13
BUN (mg/dL)	14.29 ± 3.91	14.14 ± 3.96	0.66
Cre (mg/dL)	0.81 ± 0.14	0.82 ± 0.15	1.00
eGFR (mL/min/1.73m^2^)	79.6 ± 13.91	79.13 ± 13.75	0.49
UA (μmol/l)	5.77 ± 1.25	5.98 ± 1.35	<0..05
BS (mg/dL)	94.00 ± 14.87	95.42 ± 17.28	0.09
HbA1C (mmol/mol)	5.70 ± 0.62	5.79 ± 0.59	<0.05
Amy (Units/L)	72.55 ± 36.14	72.45 ± 53.55	0.97
Urine protein level (mg/dl)	0.14 ± 0.45	0.64 ± 0.84	<0.001
Urine sugar level (mmol/L)	0.08 ± 0.53	0.13 ± 0.66	0.12

### 3.2 Analysis of physiological changes across gender

We further compared all the clinical values of pre-disaster (2017) and disaster-hit (2018) years across the gender to assess whether disaster-induced physiological differences are biased for either males or females. There was however no significant change observed for any of the given clinical values, indicating both the males or female victims have suffered health issues with almost similar magnitude (Table 2).

**Table 2:** Analysis of physiological variates compared across gender in pre-disaster (2017) and disaster-hit(2018) years.

	Pre-disaster year (2017)		Disaster-hit year (2018)		p value
	Male (N = 627) Mean ± SD	Female (N = 101) Mean ± SD	Male (N = 627) Mean ± SD	Female (N = 101) Mean ± SD	
Age (years)	49.20 ± 10.39	50.96 ± 8.67	50.20 ± 10.39	51.96 ± 8.67	0.90
Height (Cms)	169.63 ± 6.07	157.29 ± 5.72	169.88 ± 6.01	156.99 ± 5.68	0.95
Weight (Kg)	70.75 ± 12.33	56 ± 11.91	70.44 ± 11.96	55.18 ± 11.79	0.93
BMI (cells/μL)	24.56 ± 3.9	22.63 ± 4.7	24.39 ± 3.81	22.38 ± 4.61	0.84
WBC (cells/μL)	6.33 ± 1.72	5.58 ± 1.41	6.11 ± 1.57	5.22 ± 1.46	0.71
RBC (cells/μL)	4.69 ± 0.39	4.32 ± 0.35	4.79 ± 0.41	4.29 ± 0.37	0.98
Hb (Gram %)	15.35 ± 1.00	13.43 ± 1.14	15.32 ± 1.02	13.37 ± 1.19	0.80
Hct (Gram %)	46.96 ± 3.14	41.38 ± 3.33	46.15 ± 3.25	40.98 ± 3.42	0.90
MCV (fl/cell)	100.33 ± 5.39	98.02 ± 6.67	96.45 ± 5.23	95.63 ± 6.53	0.98
MCH (pg/cell)	32.81 ± 1.83	31.82 ± 2.38	32.04 ± 1.79	31.19 ± 2.4	0.87
MCHC (g/dL)	32.70 ± 0.80	32.45 ± 0.86	33.22 ± 0.81	32.61 ± 0.81	0.89
PLT (Volume %)	23.10 ± 5.69	25.19 ± 6.45	23.46 ± 5.32	23.99 ± 6.02	0.96
LDL (mg/dL)	118 ± 27.94	115.13 ± 29.44	120.24 ± 29.44	114.16 ± 27.29	0.96
HDL (mg/dL)	57.95 ± 14.33	68.94 ± 16.29	58.3 ± 14.60	70.45 ± 15.99	0.95
TG (mg/dL)	125.92 ± 98.30	88.12 ± 61.40	135.97 ± 123.46	84.44 ± 44.34	0.78
AST (Units/L)	24.73 ± 10.53	20.24 ± 5.05	25.74 ± 19.23	20.57 ± 10.5	0.88
ALT (Units/L)	27.58 ± 19.14	17.15 ± 8.45	29.62 ± 24.01	18.02 ± 15.93	0.87
ALP (Units/L)	207.56 ± 59.13	194.79 ± 81.35	211.26 ± 64.96	189.15 ± 64.13	0.79
γGTP (Units/L)	49.47 ± 63.28	26.5 ± 28.19	54.26 ± 82.42	28.47 ± 45.39	0.92
LDH (Units/L)	199.71 ± 35.53	194.86 ± 31.64	196 ± 37.47	193.89 ± 33.21	0.98
Total Protein (Units/L)	7.37 ± 0.35	7.35 ± 0.31	7.35 ± 0.38	7.27 ± 0.31	0.72
Alb (g/dL)	4.64 ± 0.26	4.45 ± 0.27	4.58 ± 0.26	4.37 ± 0.28	0.64
TBil (mg/dl)	0.82 ± 0.34	0.7 ± 0.25	0.85 ± 0.38	0.67 ± 0.24	0.26
BUN (mg/dL)	14.52 ± 3.85	12.88 ± 4.00	14.41 ± 3.95	12.44 ± 3.57	0.82
Cre (mg/dL)	0.84 ± 0.12	0.64 ± 0.11	0.85 ± 0.13	0.63 ± 0.11	0.25
eGFR (mL/min/1.73m^2^)	79.83 ± 13.90	78.16 ± 13.97	78.87 ± 13.51	80.72 ± 15.12	0.93
UA (μmol/l)	5.97 ± 1.19	4.55 ± 0.94	6.22 ± 1.24	4.47 ± 0.94	0.71
BS (mg/dL)	94.41 ± 15.04	91.43 ± 13.56	95.74 ± 17.51	93.45 ± 15.66	0.95
HbA1C (mmol/mol)	5.71 ± 0.63	5.66 ± 0.56	5.79 ± 0.6	5.79 ± 0.54	0.68
Amy (Units/L)	72.59 ± 38.03	72.33 ± 20.98	72.81 ± 56.97	70.20 ± 22.88	0.98
Urine protein level (mg/dl)	0.16 ± 0.47	0.06 ± 0.24	0.67 ± 0.87	0.40 ± 0.60	0.06
Urine sugar level (mmol/L)	0.09 ± 0.56	0.02 ± 0.20	0.14 ± 0.68	0.08 ± 0.56	0.09

### 3.3 Multivariate regression analysis

In the further step, stepwise multivariate regression with forward selection wrappers was performed to identify the association of clinical factor (s) confounding for disaster-induced deterioration of health in victims. A close perusal of the regression results (Table 3) shows that urine protein to be significantly altered (R^2^ = 0.518; step 1 of the regression analysis), indicating the victims accumulated higher urine protein in the course and following the disaster.

**Table 3:** Step wise logistic regression of physiological factors compared between pre-disaster (2017) and disaster-hit(2018) years.

Regression steps	Physiological factors	B	p value	Odds ratio at 95% C.I	R^2^
Step 1	Urine protein	-1.26	0.0001	0.28 (0.23–0.35)	0.518
	Constant	0.41	0.0001		
Step 2	Hct	0.08	0.004	1.08 (1.05–1.12)	0.526
	Urine protein	-1.31	0.0001	0.27 (0.22–0.33)	
	Constant	-3.28	0.0001		
Step 3	RBC	-0.03	0.005	0.97 (0.82–0.98)	0.547
	Hct	0.37	0.014	1.45 (1.36–1.54)	
	Urine protein	-1.31	0.0014	0.27 (0.22–0.34)	
	Constant	-2.55	0.001		
Step 4	RBC	-0.03	0.042	0.97 (0.97–0.98)	0.550
	Hb	-1.46	0.085	0.42 (0.31–1.21)	
	Hct	0.8	0.032	2.21 (1.96–2.51)	
	Urine protein	-1.36	0.002	0.26 (0.2–0.32)	
	Constant	-1.57	0.0012		
Step 5	RBC	-0.03	0.025	0.97 (0.97–0.98)	0.557
	Hb	-1.63	0.066	0.73 (0.14–1.28)	
	Hct	0.84	0.084	2.31 (0.87–2.62)	
	Alb	1.53	0.245	2.60 (0.75–3.22)	
	Urine protein	-1.34	0.002	0.26 (0.21–0.33)	
	Constant	-6.61	0.0032		
Step 12	WBC	0.14	0.057	1.15 (0.96–1.26)	0.593
	RBC	-0.03	0.024	0.97 (0.82–0.99)	
	Hb	-1.91	0.004	0.15 (0.1–0.22)	
	Hct	0.93	0.002	2.54 (2.21–2.91)	
	HDL	-0.02	0.253	0.98 (0.84–1.99)	
	γGTP	0.00	0.270	0.99 (0.79–1.73)	
	Alb	1.65	0.042	1.22 (1.07–1.87)	
	BUN	0.06	0.063	1.06 (0.81–1.53)	
	UA	-0.13	0.126	0.87 (0.78–1.95)	
	BS	0.02	0.250	1.02 (0.86–1.58)	
	HbA1C	-0.64	0.462	0.63 (0.39–1.73)	
	Urine protein	-1.45	0.001 .	0.23 (0.18–0.3)	
	Constant	-3.27	0.0021		

Regression steps those demonstrating significant regression coefficient are only presented (i.e. steps 1–5 & 12). Regression steps from 6-11 did not contribute to significant improvement of the overall regression model, hence not shown.

Year of health screening was considered as dependent variable and clinical were independent variable for the present binary logistic regression analysis.

Further, apart from a significant change in the urine protein concentration, disaster-hit changes in physiological variates like WBC, RBC, Hb, Hct, HDL, γGTP, Alb, BUN, UA, BS, HbA1C was also observed, nevertheless, even in combination, contributed considerably less to increase the overall fitness of the regression model (R^2^ = 0.593; step 12 of the regression analysis). The results of logistic regression hence suggest that, although the change in the aforementioned physiological variates impacts disaster-induced deterioration of health, however, not significantly as compared to urine protein.

### 3.4 Urine protein analysis

**Figure 2:**
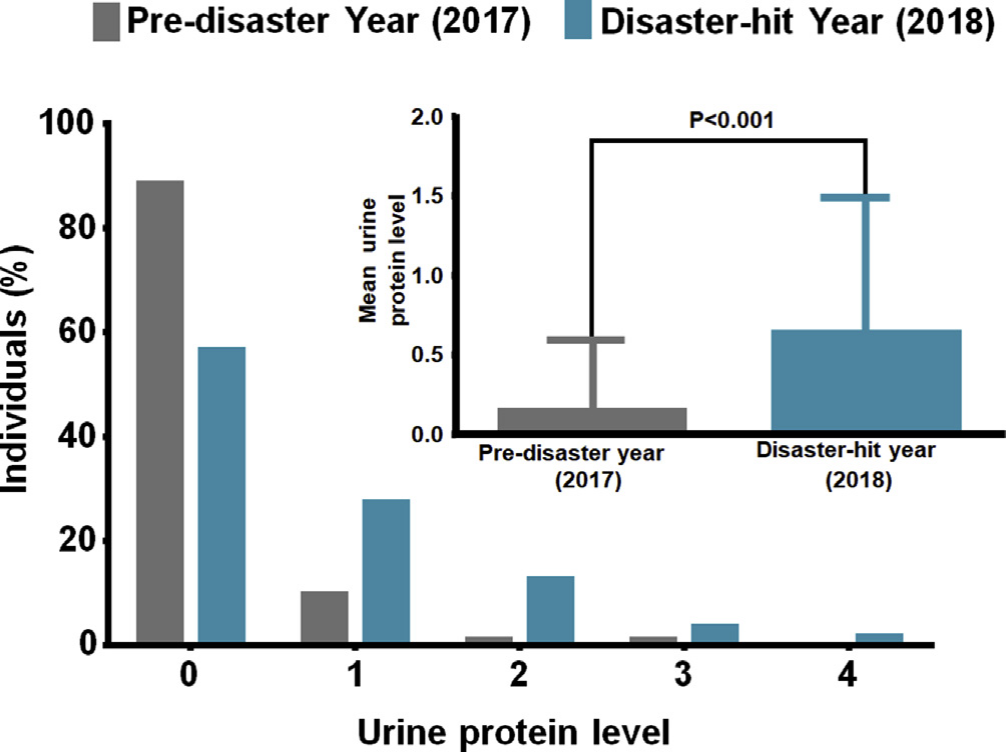
Urine protein levels in individuals. Number of individuals corresponding to varying urine protein level in pre-disaster and disaster-hit years. Inset: Mean urine protein level in individuals in pre-disaster (2017) and disaster-hit (2018) years.

Since urine protein showed a significant increase in victims due to heavy rains against pre-disaster year, we further performed a detailed logistic analysis to comprehend the extent to which it affected the victims. The levels of urine protein were defined in adherence to Japanese Committee for Clinical Laboratory Standards (JCCLS), ranging from 0 to 4, with concentration intervals corresponding to each grade as level 0 (<10 mg/dl); level 1 (10–30 mg/dl); level 2 (30–100 mg/dl; level 3 (100–300 mg/dl) and level 4 (>300 mg/dl). It was interesting to note that, in the disaster-hit year, only 56.45 % out of 728 individuals had normal urine concentration levels (Level 0), as against 88.46 % in the pre-disaster year. Further, the mean concentration of the urine protein abruptly increased by 4.5 fold in victims following the disaster (Table 4 and Figure 2).

**Table 4:** Number of individuals corresponding to varying urine protein level in pre-disaster and disaster-hit years.

Urine protein Level	Pre-disaster Year (2017) n (%)	Disaster-hit Year (2018) n (%)
0	644 (88.46)	411 (56.45)
1	70 (9.62)	199 (27.33)
2	7 (0.96)	92 (12.63)
3	7 (0.96)	25 (3.43)
4	0 (0)	1 (0.13)
Mean ± SD∗	0.14 ± 0.45	0.64 ± 0.84

∗p value < 0.01.

**Figure 3:**
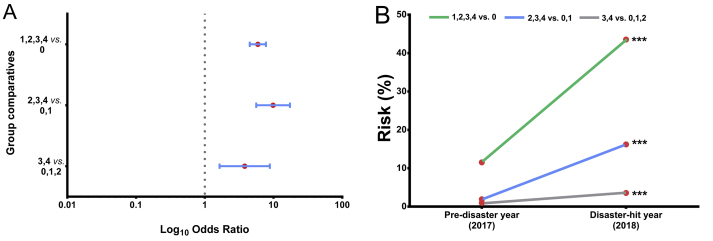
Disaster-induced risk for high protein levels. (A) Forest plot showing significant risk for high protein levels in individuals in 2018 relative to 2017 (B) Increase in the urine protein level in 2018 relative to 2017 poses a significant risk in victims.

In a further approach, we performed logistic regression comparing the normal levels of urine protein (level 0) with increased levels (levels 1, 2,3 and 4) in victims. As anticipated, higher levels of urine protein levels were found in the disaster-hit year (Table 5& Figure 3A). This is a clear indication that during the disaster, victims had to confront a severe shortage of water along with damaged sewerage pipelines in households, which in turn restricted them to use restrooms, eventually forcing them for infrequent urination. Therefore, presumably, infrequent urination may have enhanced urine protein concentration in victims thus increasing their health risk by significant fold (Table 5 & Figure 3B) during and following the disaster.

**Table 5:** Distribution of individuals into urine protein levelsinto pre-disaster and disaster-hit years.

Urine protein levels	Disaster-hit (2018) n (%)	Pre-disaster (2017) n (%)	χ2	Odds Ratio (95 % C.I.)	p value	Risk in individuals 2018 (%)	Risk in individuals 2017 (%)	Risk Ratio
4	1 (0.14)	0 (0)	0	--	--	100	49.97	2.01
0,1,2,3	727 (99.86)	728 (100)						
3,4	26 (3.57)	7 (0.96)	10.05	3.81 (1.64–8.84)	<0.05	3.57	0.96	3.71
0,1,2	702 (96.43)	721 (99.04)						
2,3,4	118 (16.21)	14 (1.92)	88.38	9.86 (5.61–17.35)	<0.01	16.21	1.92	8.42
0,1	610 (83.79)	714 (98.08)						
1,2,3,4	317 (43.54)	84 (11.54)	185.2	5.91 (4.51–7.75)	<0.01	43.54	11.54	3.77
0	411 (56.46)	644 (88.46)

We speculated that since renal anomalies/failure may be indicative of high protein level [17, 18, 19], we therefore compared the values of Estimated Glomerular Filtration Rate (eGFR) and Creatinine for both pre and disaster-hit years. However, we did not observe any significant changes suggesting victims did not suffer kidney damage during the disaster (Table 1). Furthermore, we did not identify any significant association of eGFR and Creatinine levels in enhancing urine protein levels in the disaster-hit year (Supplementary information S1 Table 2).

## 4 Discussion

According to, World Risk Report, 2016, Japan happens to be one of the world's most disaster-prone countries with the history of the 10 worst natural disasters of the 21st century [20]. Two out of the five most expensive natural disasters in recent years have occurred in Japan, costing $181 billion in the years 2011 and 1995 alone [21, 22, 23]. The types of natural disasters in Japan include earthquakes, volcanic eruptions, tsunamis, typhoons, etc. The country has tremendously suffered many years of natural disasters, affecting its economy, development, and social life [24].

Despite Japan implementing the world's most advanced disaster management system to combat earthquake-related catastrophes, efficient management for floods and heavy rain-related disasters have always been less common [25]. Till date, Nagasaki floods of 1982 was the major freshwater disaster that claimed 358 lives and forcing thousands of them homeless. It has however been past 3 decades that Nagasaki disaster struck, and in this long course of years, people have indeed witnessed earthquakes but, floods were almost rare [6]. This eventually led people for efficient preparedness for earthquakes however, not for heavy rain disasters. As there are seemingly several strategies to manage the earthquakes, the crisis management, however fell short in 2018 disaster that probably may have led to large scale casualties.

In the view of above, the present investigation is sought to assess the impact of heavy rain disaster on the health of victims and the way it afflicted their lives during and following the disaster. The present investigation was sought to analyze the health status of a significant number of 728 disaster-hit victims, the results of which may grossly reflect a similar state-of-health of Innoshima population (and for all those who have not been recruited for the study but suffered the disaster). The study herein is a comparative analysis of the health condition of individuals having health data obtained from an annual health check programs for pre-disasterand disaster-hityears.

In the present study, we assessed results of various clinical tests for organ function, (like kidney, liver) and basic metabolism, all of which were examined from blood and urine samples. As shown in Table 1, most of the clinical variates had an insignificant change in victims after the catastrophe. However, RBC, Hct, MCV, MCH and MCHC, Albumin, Uric acid, and importantly urine protein demonstrated a significant change. The results thus clearly imply, the heavy rains brought about serious deterioration of health in victims.

Of all the clinical factors that have shown significant changes, there was a sharp rise in urinary protein levels in victims soon after the disaster. We found that there were remarkably higher levels (levels ranging from 3-4) of urine protein in same individuals in the disaster-hit year, who otherwise had normal urine levels a year before the disaster (Levels ranging from 0-2).

We further surged to identify the rationale for such high levels of urinary proteins in victims. In general physiological considerations, dehydration causes a significant increase in urinary protein levels [26, 27]. Given that water outage is usual to any natural disasters, the victims generally suffer from dehydration due to a crisis of drinking water. It was therefore assumed that dehydration would have probably led to higher urine protein concentrations in the victims in the present study too. Interestingly, if dehydration, would have been the plausible rationale for higher urine protein as anticipated, the effect of dehydration should have also caused a decline in the levels of RBC, HCT, Hb, MCV, MCH, and MCHC. Contrastingly, these aforementioned levels significantly enhanced in victims for disaster-hit year (the reason for which remains elusive). Therefore, the above results suggesting dehydration as a sole confounding factor for enhanced urine protein levels cannot be justified in the present study.

The most prominent physiological elucidation for high urine protein levels can indicate infrequent urination. In this regard, we further sought for water outage in the Innoshima households which aided us to identify the basis for heightened protein levels in victims. According to the Disaster Management Agency of Hiroshima Prefecture, and Hitachi Zosen Health Insurance Association, Innoshima, a total of 59,844 households were hit with water outage on the first day of the disaster (7 July 2018) and only 23.3 % of the households were recovered from outage in a week, while 69.7 % of households were recovered after 10 days of the heavy rain. The entire water restoration process in all 59,844 households was established in 2 weeks (20 July 2018) (the complete water outage report is provided in the supplementary information S1 Table 1). This extremely long span of water crisis forced people to refrain from using restrooms or exercising other sanitary activities. Moreover, victims solely depended on the drinking water and preserved it by evading the use of toilets. Adding to the difficulty, the sewerage pipelines were extensively damaged during the disaster, which further made victims avoid restrooms. This two weeks of troublesome living of victims by avoiding restroom well indicates infrequent urination, which further justifies their increased urine concertation of protein.

Furthermore, as suggested by previous studies, infrequent urination has also been attributed to enhanced uric protein levels in patients [28, 29, 30], a similar observation can be noticed in the present study too. Therefore, from the present study, we state that although dehydration may not be associated to an increase in the urine concentration, the infrequent urination during the water outage may form a strong confounding factor for increased urine protein levels in victims following the disaster. In addition, dehydration not forming the main confounder for elevated urine concentrations can be attributed to the fact that, municipal corporations of Onomichi and Fukuyama City aided by Japan Self Defence forces ensured to provide safe drinking water which was delivered through an articulated water tanker in each area. Victims in fact, solely depended on the drinking water and preserved it by evading the use of toilets. Although there was an acute water outage in the households (due to damaged pipelines), which restricted residents to carry out any water-related chores (use of restrooms, washing etc), the drinking water was however available. In addition, since the clean drinking water was available for victims, it further rules out the possibility of waterborne illnesses like gastroenteritis to have increased urine protein concentration. This observation further supports the fact that, infrequent urination may be the most important factor for elevated protein levels in victims.

It may well be thought that there may be a gradual decrease in the urine protein levels (caused by infrequent urination) after the water outage period and would only be irreversible unless any medical condition associated with it. In addition, and for the fact that, in the disaster-hit year in 2018, the annual health screening was conducted for 1.5 months, the individuals screened after the 1st week of disaster can be expected to have higher urine protein concentration than those screened at the 8th week following the disaster. However, we did not find any significant change in the mean urine protein level in a group of individuals who visited at the later weeks of disaster compared to the first week (Supplementary information S1 Tables 3 and 4). This observation indicates that the urine protein concentration was not much affected in individuals within 1.5 months of the disaster. Therefore, what may be possibly assumed that the urine protein level to regress to normal levels considerably takes more time, which may be above 1.5–2 months. Therefore, in order to understand whether urine protein levels decreased (in the absence of medical condition), a gap of 1 year may be more appropriate. To pursue this, we analyze how urine protein levels changed in individuals soon after the disaster i.e. by comparing the levels between 2017 to 2018 and a year after the disaster i.e. 2019. A keen perusal on the comparison showed that the urine protein levels were elevated in 39 % of the individuals soon after the disaster (2018), however, elevated levels were observed in only 14.8 % of the individuals a year after the disaster (2019) (Supplementary information S1 Table 5 & Figure 1). Further, most of the victims who otherwise showed higher urine protein levels soon after the disaster in 2018, had almost normal levels of urine protein in 2019, which indicates that they have recovered from higher urine protein levels in the span of 1 year (Supplementary information S1 Table 6). Interestingly, when urine protein levels were compared between 2017 and 2019 (i.e a year before the disaster and a year after the disaster), the difference of mean urine protein is insignificant which is in contrast to a significant increase in urine protein upon comparison to 2017 to 2018 (Supplementary information S1 Table 7 & Figure 2). Hence, this indicates that; had individuals suffered from chronic illness and systemic diseases (although we have not registered such cases for the study), there would not have been a decrease in the urine protein levels a year after the disaster, but in contrast in the present scenario, a significant decline in the urine protein levels can be observed. Therefore, these observations indicate that the observed increase in urine protein is not pathological instead is disaster-induced.

Most of the disaster influenced health studies adopts a retrospective approach wherein the health effects of the victims are analysed following the aftermaths of the disaster [31, 32, 33, 34, 35]. The present investigation, in contrast, is prospective in manner, which involves a comparison of state-of-health of individuals at pre-and disaster-hit periods. This approach helps one to understand the magnitude to which the health deteriorated particularly attributing to the disaster thereby excluding other probable causes. In addition, there is limited empirical evidence to show the consequence of water outage on the health condition in disaster-affected victims, while, the present study, has attempted to rationally address the significant impact of water outage leading to potential health problems like the case of increase in the urine protein.

To summon, the purpose of the present study is to convey the perils that people of Innoshima sustained during the disaster particularly in the period of water outage. The outcomes of this investigation holds particular relevance and may help the disaster management council and health care providers to device efficient monitoring strategies for mitigating health issues in the future events of disaster.

## 5 Conclusion

The reports of the Health Protection Agency (HPA) based on enormous disaster-related literature have identified that disasters have uprooted civilizations, brought profound loss to economy, but most importantly it has affected the health of a person at both physiological and psychological dimensions. The present study stands as testimony at the extent to which disasters can influence the health of the people. The increase in urine protein concentration in otherwise healthy individuals due to water outage during the disaster, is indeed a serious issue which needs to be meticulously addressed, because the findings of the present study may hold relevant to future disasters as well. Hence, it becomes indispensable to establish a combined strategy by concerted efforts of disaster management agencies, aftercare health services, and policymakers to address these issues and help in improving the resilience of people to cope with disasters in future.

## Declarations

### Author contribution statement

T. Ito: Conceived and designed the experiments; Performed the experiments; Wrote the paper.

S. Bandaru: Performed the experiments; Analyzed and interpreted the data; Wrote the paper.

Y. Shimizu: Performed the experiments; Analyzed and interpreted the data.

Y. Seki, T. Sasaki, H. Wada and T. Otsuki: Analyzed and interpreted the data.

Y. Okano: Contributed reagents, materials, analysis tools or data.

### Funding statement

This research did not receive any specific grant from funding agencies in the public, commercial, or not-for-profit sectors.

### Competing interest statement

The authors declare no conflict of interest.

### Additional information

No additional information is available for this paper.

## Appendix A Supplementary data

SUPPLEMETARY INFORMATION SUPPLEMETARY INFORMATION
