# Supplementary material for: Impact of heavy rains of 2018 in western Japan: disaster-induced health outcomes among the population of Innoshima Island
Source: Heliyon. 2020 May 25;6(5):e03942. doi: 10.1016/j.heliyon.2020.e03942 (PMC7256463; doi:10.1016/j.heliyon.2020.e03942)
Supplement: SUPPLEMETARY INFORMATION [file mmc1.docx]

**Supplementary information**

**Impact of heavy rains of 2018 in Western Japan: Disaster-induced health outcomes among the population of Innoshima Island.**

**Srinivas Bandaru^1^, Shunji Sano^2^, Yurika Shimizu^1, 3^, Yuka**

**Seki^1^, Yoshikazu Okano^4, 5^, Tamaki Sasaki^6^, Hideho Wada^7^,**

**Takemi Otsuki^8^, Tatsuo Ito^1, 2*^**

^1^Department of Public Health, Okayama University Graduate School of Medicine,

Dentistry, and Pharmaceutical Sciences, Okayama, Okayama, Japan

^2^Department of Surgery, Division of Pediatric Cardiothoracic Surgery, University of

California San Francisco, San Francisco, CA, USA

^3^Department of Pathophysiology - Periodontal Science, Okayama University Graduate

School of Medicine, Dentistry, and Pharmaceutical Sciences, Okayama, Okayama, Japan

^4^Hitz Hitachi Zosen Health Insurance Association Clinic at Innoshima, Onomichi,

Hiroshima, Japan

^5^Innoshima General Hospital, Onomichi, Hiroshima, Japan

^6^Department of Nephrology & Hypertension, Kawasaki Medical School, Kurashiki,

Okayama, Japan

^7^Department of Hematology, Kawasaki Medical School, Kurashiki, Okayama, Japan

^8^Department of Hygiene, Kawasaki Medical School, Kurashiki, Okayama, Japan

*** Author of Correspondence (TI)**

[tataito@okayama-u.ac.jp](mailto:tataito@okayama-u.ac.jp)

**Table 1.** Water outage in households of Innoshima during heavy rain and recovery rates. (Source: Hitachi Zosen Health Insurance Association, Innoshima, Japan).

| **Date**  **( All the days in July 2018 )** | **Households with water outage**  **(N)** | **Households recovered from water outage**  **(N)** | **Total households recovered from water outage**  **(N)** | **Recovered Households (%)** |
| --- | --- | --- | --- | --- |
| 7 | 59844 | 0 | 0 | 0 |
| 11 | 50344 | 9500 | 9500 | 15.9 |
| 13 | 47424 | 2920 | 12420 | 20.8 |
| 15 | 45924 | 1500 | 13920 | 23.3 |
| 16 | 24424 | 21500 | 35420 | 59.2 |
| 17 | 18124 | 6300 | 41720 | 69.7 |
| 18 | 4124 | 14000 | 55720 | 93.1 |
| 19 | 1124 | 3000 | 58720 | 98.1 |
| 20 | 0 | 1124 | 59844 | 100 |

**Table 2.** EGFR and creatinine levels stratified into urine protein levels in pre- disaster and disaster-hit years

|  | **Urine Protein levels** | **2017** | **2018** | **p value** | **OR at 95 % CI** |
| --- | --- | --- | --- | --- | --- |
| **eGFR** | 0 | 644(80.21±13.83) | 411(80.48±14.03) | ref |  |
|  | 1 | 70(75.07±13.52) | 199(79.59±12.07) | 0.89 | 1.05 (0.67-1.64) |
|  | 2 | 7(72.23±11.78) | 92(73.38±13.34) | 0.95 | 1.01 (0.64-1.58) |
|  | 3 | 7(75.63±18.38) | 25(72.76±16.87) | 0.94 | 0.95(0.61-1.49) |
|  | 4 |  | 1(75.4) | --- |  |
| **Creatinine** | 0 | 644(0.8±0.13) | 411(0.8±0.14) | ref |  |
|  | 1 | 70(0.86±0.15) | 199(0.8±0.14) | 0.28 | 0.93 (0.01-71.64) |
|  | 2 | 7(0.88±0.12) | 92(0.89±0.15) | 0.27 | 1.01 (0.01-72.76) |
|  | 3 | 7(0.9±0.21) | 25(0.92±0.2) | 0.28 | 1.02 (0.01-71.52) |
|  | 4 |  | 1(0.92) | --- |  |

**Table 3.** Attendance of individuals for health screening following the disaster (2018)

| **Date (From -To)** | **Weeks following the disaster** | **No . of participants attended health screening** | **Mean Urine protein concentration** | **p value** |
| --- | --- | --- | --- | --- |
| 19 July - 21 July | 2 | 22 | 0.36±0.66 | ref |
| 22 July - 28 July | 3 | 86 | 0.64±0.85 | 0.103 |
| 29 July - 4 Aug | 4 | 281 | 0.59±0.81 | 0.134 |
| 05 Aug - 11 Aug | 5 | 155 | 0.7±0.86 | 0.051 |
| 12 Aug-18 Aug | 6 | 91 | 0.68±0.94 | 0.062 |
| 19 Aug-25 Aug | 7 | 67 | 0.72±0.81 | 0.048 |
| 26 Aug-02 Sep | 8 | 26 | 0.58±0.86 | 0.322 |

**Table 4.**  Stratification of individuals based on urine protein levels recorded for their attendance in a given week following the disaster.

|  | **Urine protein level** | | | | |  |
| --- | --- | --- | --- | --- | --- | --- |
| Weeks following the disaster | **0** | **1** | **2** | **3** | **4** | **Total no. of individuals screened in a week** |
| 2 | 16 | 4 | 2 |  |  | 22 |
| 3 | 48 | 24 | 12 | 1 | 1 | 86 |
| 4 | 166 | 72 | 35 | 8 |  | 281 |
| 5 | 82 | 43 | 25 | 5 |  | 155 |
| 6 | 52 | 23 | 9 | 7 |  | 91 |
| 7 | 31 | 27 | 6 | 3 |  | 67 |
| 8 | 16 | 6 | 3 | 1 |  | 26 |
| **Total** | **411** | **199** | **92** | **25** | **1** |  |

The original data comparing pre- and disaster-hit years (2017 vs 2018 respectively) comprised 728 participants, however, only 711 of them were available in 2019 (as some of the employees retired, transferred or have not attended the health screening). The comparison for urine protein levels, therefore, has been conducted only for 711 individuals for 2017, 2018 and 2019 as shown in the following tables 5, 6 and 7.

**Table 5.** Change in urine protein levels in individuals in a span of one year

| **Change in urine protein levels** | **2017 to 2018**  **N(%)** | **2018 to 2019**  **N(%)** | **p value** |
| --- | --- | --- | --- |
| Unchanged | 393(55.3) | 443(75.8) |  |
| Elevated | 277(39.0) | 31(14.8) | <0.001 |
| Decreased | 41(5.8) | 237(9.4) | <0.001 |


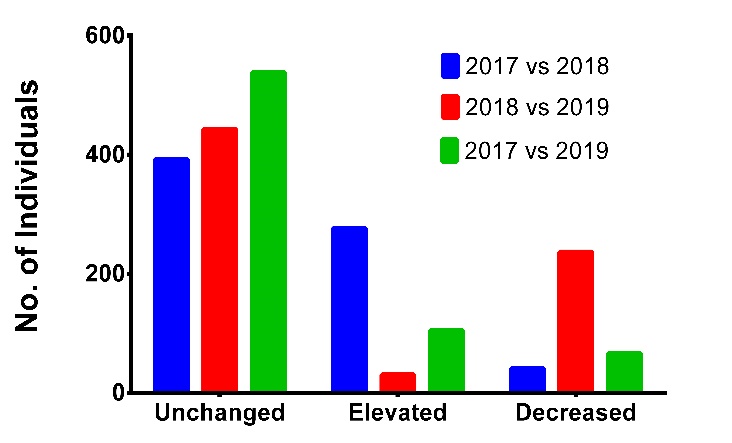


**Figure 1.** Change in urine protein in individual’s levels in a span of one year

**Table 6.** Number of individuals showing change in urine protein levels in comparison to 2017 and 2018 as well as 2018 and 2019.

| **Year** | **urine protein levels** | **0** | **1** | **2** | **3** | **4** | **Total** |
| --- | --- | --- | --- | --- | --- | --- | --- |
| **2017 ↓ *vs.* 2018→** | 0 | 367 | 171 | 74 | 18 | 0 | 630 |
|  | 1 | 36 | 21 | 11 | 0 | 0 | 68 |
|  | 2 | 2 | 1 | 2 | 2 | 0 | 7 |
|  | 3 | 0 | 1 | 1 | 3 | 1 | 6 |
|  | 4 | 0 | 0 | 0 | 0 | 0 | 0 |
|  | **Total** | 405 | 194 | 88 | 23 | 1 |  |
| **2018 ↓ *vs.* 2019→** | 0 | 383 | 18 | 4 | 0 | 0 | 405 |
|  | 1 | 141 | 49 | 4 | 0 | 0 | 194 |
|  | 2 | 58 | 18 | 7 | 5 | 0 | 88 |
|  | 3 | 5 | 12 | 2 | 4 | 0 | 23 |
|  | 4 | 0 | 0 | 1 | 0 | 0 | 1 |
|  | **Total** | 587 | 97 | 18 | 9 | 0 |  |
| **Legend:** |  |  |  |  |  |  |  |
|  |  | Elevated |  |  |  |  |  |
|  |  | Unchanged |  |  |  |  |  |
|  |  | Decreased |  |  |  |  |  |

**Table 7.** Comparison of urine protein levels in individuals recorded in 2017, 2018 and 2019.

| **urine protein levels** | **2017** | **2018** | **2019** | **p values** | | | | | |
| --- | --- | --- | --- | --- | --- | --- | --- | --- | --- |
|  |  |  |  | **Year wise comparison of each level** | | | **Year wise comparison of all levels** | | |
|  |  |  |  | **2017 vs 2018** | **2018 vs 2019** | **2017 vs 2019** | **2017 vs 2018** | **2018 vs 2019** | **2017 vs 2019** |
| 0 | 630(88.61) | 405(56.96) | 587(82.56) | ref | ref | ref | <0.001 | <0.001 | 0.014 |
| 1 | 68(9.56) | 194(27.29) | 97(13.64) | <0.001 | <0.001 | 0.01 |  |  |  |
| 2 | 7(0.98) | 88(12.38) | 18(2.53) | <0.001 | <0.001 | 0.03 |  |  |  |
| 3 | 6(0.84) | 23(3.23) | 9(1.27) | <0.01 | <0.01 | 0.51 |  |  |  |
| 4 | (0) | 1(0.14) | (0) | -- | -- | -- |  |  |  |
| Mean±SD | 0.14±0.44 | 0.62±0.83 | 0.23±0.55 |  |  |  |  |  |  |


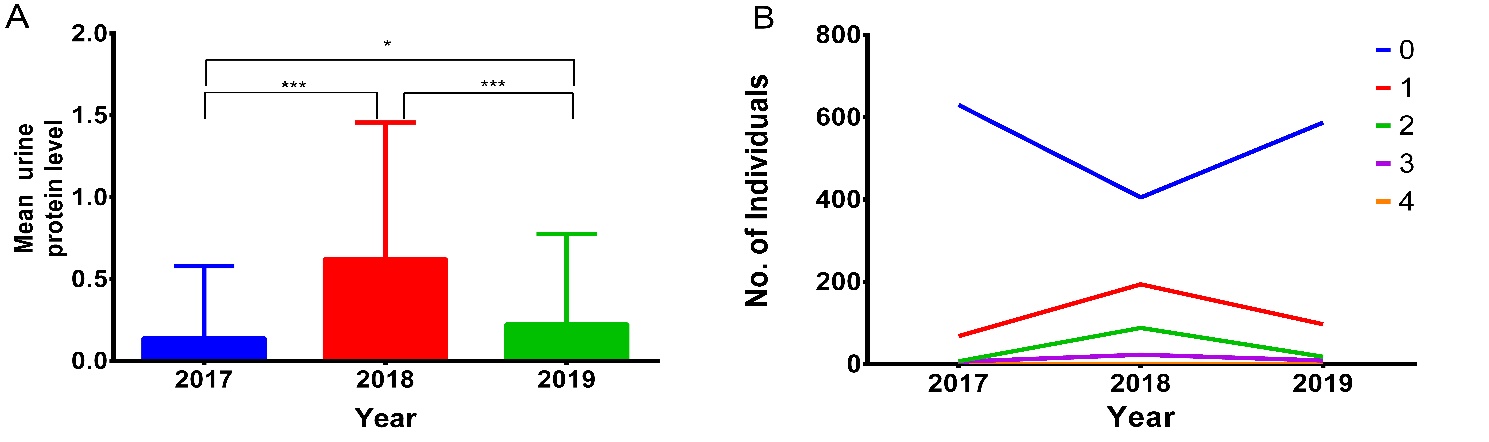


**Figure 2.** **(A)** Mean urine protein levels in individuals for three consecutive years. **(B)** Number of individuals showing change in urine protein levels for three consecutive years.
